# Life cycle environmental sustainability and cumulative energy assessment of biomass pellets biofuel derived from agroforest residues

**DOI:** 10.1371/journal.pone.0275005

**Published:** 2022-10-07

**Authors:** Ahmad Rashedi, Niamat Gul, Majid Hussain, Rana Hadi, Nasreen Khan, Sayyada Ghufrana Nadeem, Taslima Khanam, M. R. M. Asyraf, Virendra Kumar

**Affiliations:** 1 College of Engineering, IT & Environment, Charles Darwin University, Ellengowan Drive, Casuarina, Northern Territory, Australia; 2 Department of Forestry and Wildlife Management University of Haripur, Khyber Pakhtunkhwa, Pakistan; 3 Department of Zoology, Jinnah University for Women, Karachi, Sindh, Pakistan; 4 Department of Microbiology, Jinnah University for Women, Karachi, Sindh, Pakistan; 5 Institute of Energy Infrastructure, Universiti Tenaga Nasional, Jalan IKRAM-UNITEN, Kajang, Selangor, Malaysia; 6 Department of Mechanical Engineering, Harcourt Butler Technical University, Kanpur, India; University of Huddersfield, UNITED KINGDOM

## Abstract

This study was carried out to produce low-emitting biomass pellets biofuel from selected forest trees such as (*Cedrus deodara and Pinus wallichiana*) and agricultural crop residues such as (*Zea mays and Triticum aestivum*) in Gilgit-Baltistan, Pakistan using indigenously developed technology called pelletizer machine. Characterization, environmental life cycle impact assessment, and cumulative energy demand of biomass pellets biofuel produced from selected agriculture crops and forest tree residues were conducted. The primary data for biomass pellets production was collected by visiting various wood processing factories, sawmills, and agricultural crop fields in the study area. Biomass pellets are a type of biofuel that is often made by compressing sawdust and crushing biomass material into a powdery form. The particles are agglomerated as the raw material is extensively compressed and pelletized. Biomass pellets have lower moisture content, often less than 12%. Physically, the produced pellets were characterized to determine moisture content, pellet dimensions, bulk density, higher heating value, ash content, lower heating value, and element analysis. A functional unit of one kilogram (kg) biomass pellets production was followed in this study.The life cycle impact assessment of one kg biomass pellets biofuel produced from selected agro-forest species revealed environmental impact categories such as acidification (0.006 kg SO2 eq/kg pellets), abiotic depletion (0.018 kg Sb eq/kg pellets), marine aquatic ecotoxicity (417.803 kg 1,4-DB eq/kg pellets), human toxicity (1.107 kg 1,4-DB eq/kg pellets), freshwater aquatic ecotoxicity (0.191 kg 1,4-DB eq/kg pellets), eutrophication (0.001 kg PO4 eq/kg pellets), global warming (0.802 kg CO2 eq/kg pellets), and terrestrial ecotoxicity (0.008 kg 1,4-DB eq/kg pellets). Fossil fuel consumption was the hotspot source to all environmental impacts investigated. To measure the cumulative energy demand of biomass pellets made from different agroforestry species leftovers showed that the maximum cumulative energy was from wheat straw pellets (13.737 MJ), followed by corncob pellets (11.754 MJ), deodar sawdust pellets (10.905 MJ) and blue pine sawdust pellets (10.877 MJ). Among the various production activities, collection and transportation of primary raw material, crushing, screening, adding adhesives, pelletizing, cooling, final screening, and packing have the maximum contribution to the water scarcity index, followed by lubricating oil (0.00147m^3^). In contrast, the minimum contribution to water footprint was from electricity (0.00008m^3^) and wheat starch (0.00005m^3^). The highest contribution to the ecological footprint impact categories such as carbon dioxide, nuclear, and land occupation was lubricating oil and less contribution of wheat starch and electricity for manufacturing one kg pellets biofuel. It is concluded that physico-mechanical and combustion properties of the biomass pellets biofuel developed in the present study were following the Italian recommended standards. Therefore, it is strongly recommended that the Government of Pakistan should introduce the renewable biomass pellets industry in the country to reduce dependency on fossil fuels for cooking and heating purposes.

## 1. Introduction

Energy is critical in the present and future of the globe. Annual global energy demand has risen steadily over the last 15 years, from 10,000 to 13,000 million tonnes equivalent to Petroleum [1, 2]. Most energy systems in developed and developing countries rely on fossil fuels [3, 4]. Fossil fuel consumption contributes to environmental issues including global warming and climate change. Both of these factors contribute to health issues and reduce people’s standard of living [5]. The need to reduce the consumption of conventional (fossil) fuels and replace them with fuels obtained from renewable energy has prompted a surge in research into biofuels derived from a renewable energy source such as biomass [6, 7]. Globally, there has been a focus on biofuels, particularly those derived from crops such as corn, sugarcane, and soybeans, which are regarded as renewable energy sources by some experts. Wood and crop leftovers are also used as a biofuel [8, 9]. Alternative energy sources that are nearby available, particularly renewable, and ecologically beneficial by helping CO_2_ balance should be investigated and used to meet energy demands [10, 11]. Forestry, agricultural leftovers, and residues are being investigated as a critical resource of bioenergy and biomaterials. Their use is tempting since they are not linked to indirectly or directly land use alters issues, and which are by-products of current processes, they are expected to be low-cost. Many economic evaluation models suggest that residues could be a key source of energy in the future, especially in a scenario with severe climate alleviation [12, 13]. Bioenergy consumption results in limited allowed land usage, less biodiversity, and other ecological impacts, and lower overall climate change [14, 15]. Biomaterials, which contain crop residuals (wheat/rice straws and maize trash), and forest residuals, will be seen as a source of sustainable and clean energy which might be given additional consideration all over the globe to ease current energy and ecological concerns [16]. The manufacture of high-density biomass pellets (BPs) might resolve the mentioned problems and its usage has expanded quickly in recent decades due to cost reductions and the fact that they are renewable [17]. The biomass production process comprises several stages in which the biomass is treated to generate compacted and densified material. The first stage is milling, which produces material with uniform particle sizes. Following that, the materials are dried, and magnets are employed to extract the metallic elements during the cleaning process. After passing through these phases, the material is moistened and forced into a pelletizer machine, which lowers the temperature and increases the hardness of the solid biofuel [18]. A compaction technique such as pelleting is required to create solid biofuels that may substitute fossil fuels such as coal from biomass. The pellets are made by subjecting the biomass material (sawdust from woody biomass, ground agricultural biomass residues, or mixes of the two, with or without additives) to high pressures and forcing it through the cylindrical holes of a flat or cylindrical die. The biomass "fuses" due to the temperature and friction forces that occur inside the machine, resulting in compact and homogenous pellets [19–21]. Pelletization is converting raw biomass into pellets with improved fuel quality, such as increased bulk density and uniform shape and size [22]. Biomass pellets biofuel are made from wood wastes such as sawdust, chips, and planer shavings. These leftovers are low in density and voluminous, making them difficult to use as fuel. As a result, it is feasible to crush them into dense pellets with a high energy content that is easy to store, carry, and ignite [23]. Wood pellets are a popular biofuel progressively replacing oil and firewood in household heating and cooking [24].

One of the most common methodologies for assessing the environmental impacts of products is life cycle assessment (LCA) [20, 25–27]. LCA is a method for examining a product’s or process’s environmental impact from inception through its final disposal. LCA is a perfect tool for analyzing the environmental impacts of biomass pellets biofuel manufacturing since this has been extensively utilized for goods creation, product upgrading, public policy formulation, and product environmental profile analysis globally [28–30]. Thus, the LCA of biomass pellets biofuel production chain in Gilgit-Baltistan, Pakistan was critical to explain the position of the ecological repercussions and then plan for a decrease of the ecological impacts in the future. Therefore, the current study’s objectives were to first, produce biomass pellets biofuel from agroforest residues and then evaluate pellet properties, taking into account energy (calorific value and combustibility index), physical properties (length, diameter, moisture absorption, and bulk density), mechanical properties (compression resistance and durability), and other parameters (ash and moisture content) of four types of biomass pellets biofuel manufactured from agriculture and forestry crops (*Zea mays*, *Triticum aestivum*, *Cedrus deodara*, *and Pinus wallichiana*) residues, respectively and Finally, to conduct a comprehensive LCA for measuring environmental footprint and cumulative energy demand of biomass pellets biofuel derived from agriculture and forest crops residues in Gilgit-Baltistan, Pakistan.

## 2. Materials and methods

### 2.1. Description of the study area

**Fig 1:**
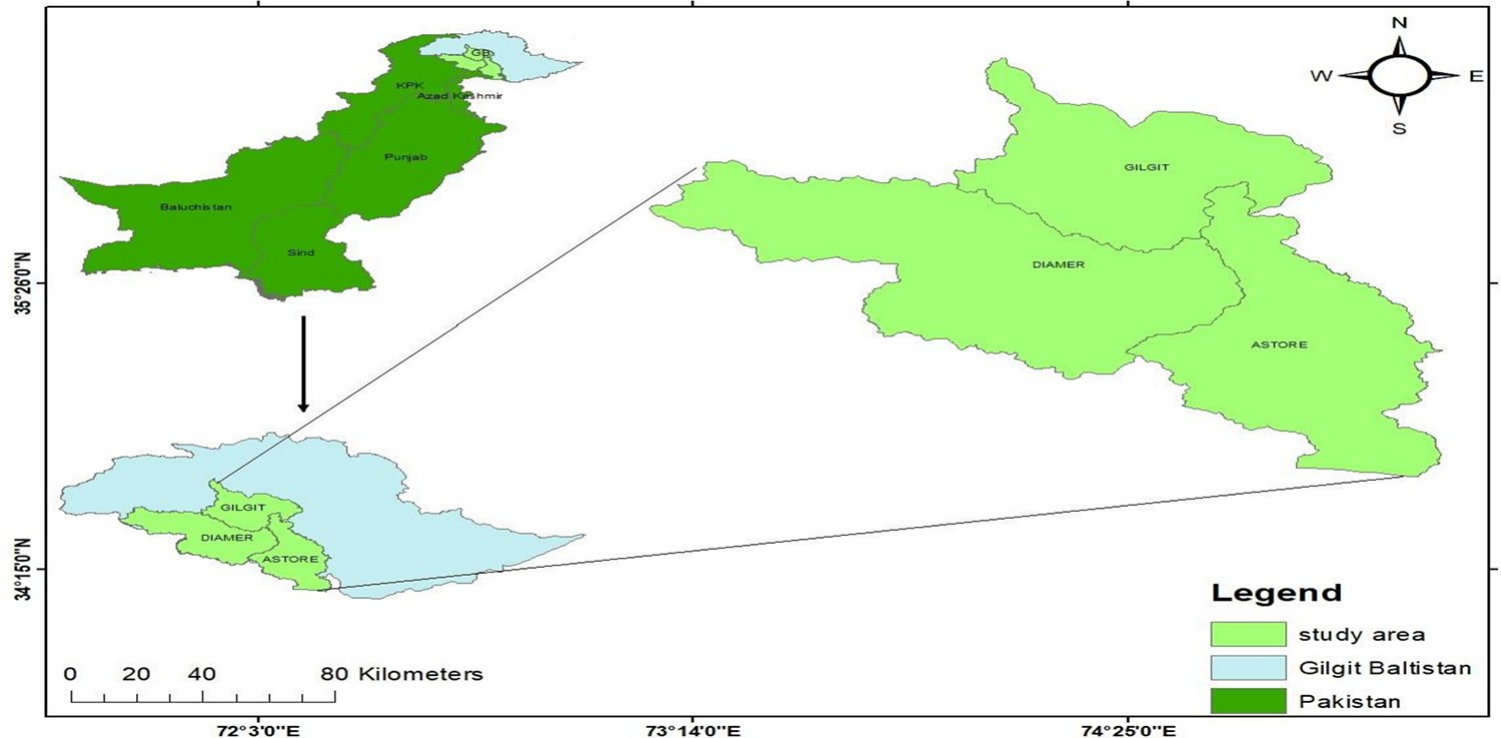
Location map of the study area.

The newly constituted province of Gilgit-Baltistan is located in Pakistan’s northern areas, near the Indian, Chinese, and Afghan border belts. Gilgit Baltistan has an area of 72496 square kilometers (km^2^) and is located between 35.8026° North longitude and 74.9832° East latitude. It stretches from the Hindukush in the north to the Karakorum in the east, with the Western Himalaya in the south and the Pamirs in the far north. These significant mountain ranges meet at a crossroads. With a surface area of roughly 27,188 square miles, Gilgit-Baltistan is predominantly a country of trans-Himalayan nature, with few cis-Himalayan characteristics, monsoon rains, and plains seasons [31]. The current study was conducted in the Gilgit-Baltistan province of Pakistan as shown in Fig 1. Gilgit-Baltistan is primarily covered with dry temperate woodlands. Various coniferous trees, such as kail, deodar, blue pine, and spruce are abundant. The entire vegetation cover in Gilgit-Baltistan is 2849 km^2^, of which 2196 km^2^ are privately held by the tribal populations of Tangir, Darel, and Chilas in the Diamer district. The district’s private forest cover is estimated to be 30%. Wheat and maize are the major agricultural crops cultivated in the districts of Diamer, Astore, and Gilgit compared to other districts of Gilgit-Baltistan, Pakistan. It may be cultivated not only in tropical and sub-tropical zones but also in temperate zones and frigid northern tracts, even beyond 60 degrees north latitude.

### 2.2. Laboratory work

For pellets biofuel prototype production, biomass samples were passed through different stages in the laboratory, such as; material crushing, sieving, drying, and adding chemicals. In the laboratory to manufacture biomass pellets biofuel from agroforestry residues using indigenously developed pelletizer technology. To demonstrate that biomass pellets are one of the best biofuels compared to other fossil fuels like coal, fossil fuels, etc.

### 2.3. Production process of biomass pellets

#### 2.3.1. Biomass sample collection

**Fig 2:**
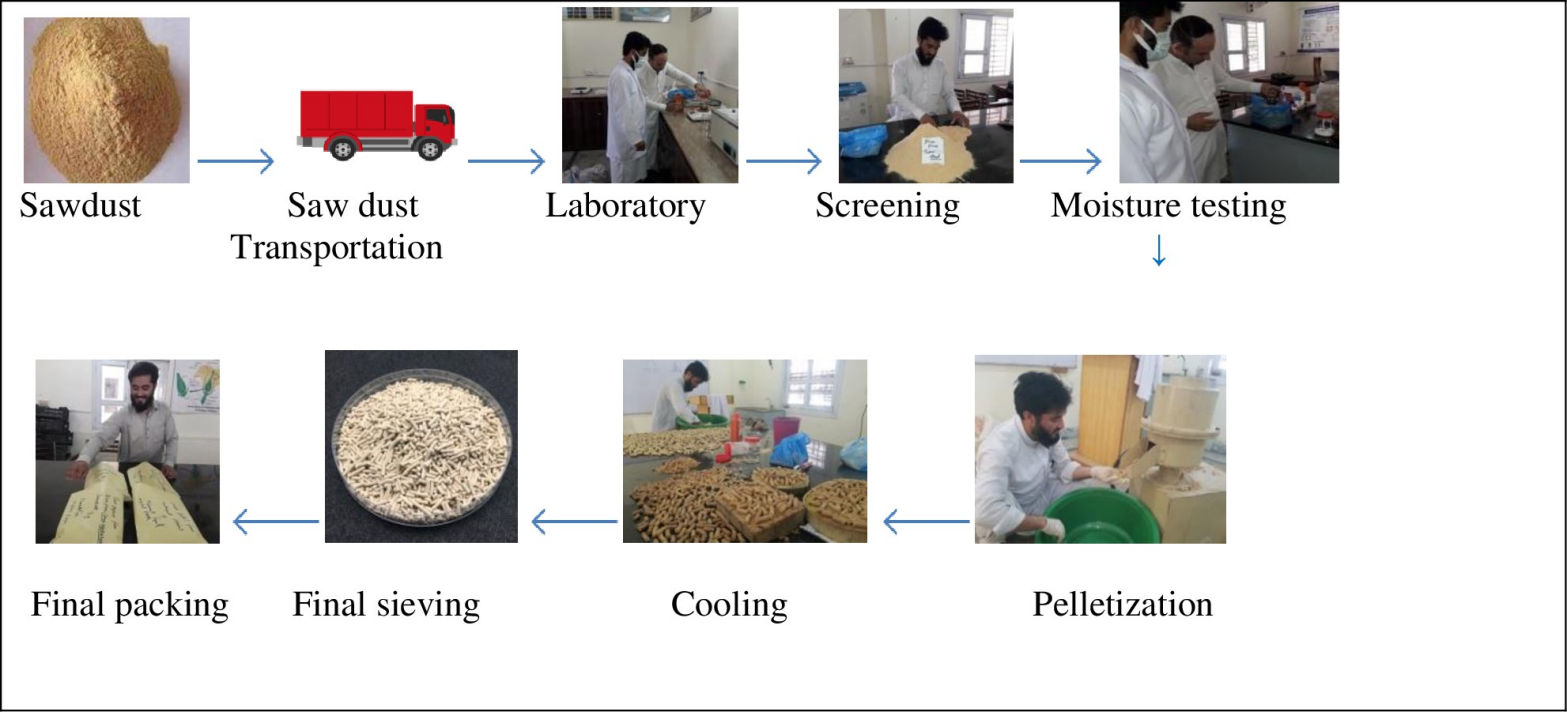
System boundary of the present study.

The current research study was carried out on producing biofuel pellets from agriculture and forestry residues using an indigenously developed technology pelletizer machine. Biomass samples were collected by visiting agricultural sites in the study area in Gilgit Baltistan such as agricultural crop fields, furniture shops, and sawmills. These samples of raw materials of biomass were brought to the University of Haripur Industrial Forestry and Wood Technology Laboratory for biomass pellets production. For the sampling, we selected two softwood tree species sawdust; *Cedrus deodara* (Deodar), *Pinus wallichiana* (Blue pin), and two agriculture crops residues; *Zea mays* (Corncob) and *Triticum aestivum* (wheat straw) grown at Gilgit-Baltistan province of Pakistan as can be seen in Fig 2.

#### 2.3.2. Grinding

When the raw materials are ready, the first step is to reduce the size of the dry raw materials. The raw materials were ground through a hammer mill during the grinding process, which crushed them into smaller pieces less than 5mm in diameter. After the grinding process, the biomass raw materials attained the requisite size and moisture content for biomass pellet biofuel manufacturing. The raw materials used to make biomass pellets must have less than 5 mm to produce pellets with good durability. These two materials like blue pine sawdust and deodar sawdust are available in crushed form (with a diameter less than 5mm) from wood processing factories and chain sawmills. The raw materials of two agriculture crops samples i.e., corncob and wheat straw are needed to crush through a hummer or grinder to convert them into the required diameter size of less than 5mm. For this purpose, a chipper machine was used to reduce the size of the agricultural crop’s residues to up to 40mm size. Then a hammer mill machine was used to further reduce its size to less than 5mm diameter, which is recommended materials size for biomass pellets biofuel manufacture.

#### 2.4.3. Screening

After creating chips or sawdust, the raw materials were sieved and screened to remove contaminants like stone and metal particles from the dried biomass chips. These contaminants can cause the pelletizer machine to fail mechanically.

#### 2.4.4. Additives

Sporadically a binding agent bio binder, lubricating oil, and water are mixed with the samples to develop high-quality biomass pellets biofuel. Additives with beneficial qualities are frequently used e.g., starch and lignin. However, it is well-intentioned to highlight that it is not always essential to use additives; subsequently, it will perhaps provide undesirable substances and as a result, will contribute to higher ash content and upsurge the cost of manufacturing biomass pellets.

#### 2.4.5. Pelletizing

After the grinding and screening process, the biomass raw materials attained the requisite size and moisture content for biomass pellet biofuel manufacturing. As a result, the raw materials were pelletized into biomass pellets biofuel. Pelletizer is a machine that converts raw materials into biomass pellets biofuel through the process of densification under high heat and pressure. Before going to the dies, raw materials must be processed with water and other additives in a mixing chamber to sort the soft and hot materials.

#### 2.4.6. Cooling

After pellets biofuel production, cool the pellets to room temperature, because cooling not only removes moisture formed by heated pellets during the densification stage but also improves the storage stability of these biomass pellets. The most generally used cooling equipment is based on countercurrent airflow in the pellet’s flow. In our study ceiling fans were used to air-cooled the biomass pellets biofuel in the Industrial Forestry and Wood Technology Lab of the University of Haripur, Pakistan.

### 2.5. Life Cycle Assessment (LCA)

The compilation of inputs, outputs, and associated ecological burdens caused by a product system during its entire lifecycle stages is known as LCA [32, 33]. LCA is a tool for assessing a product’s or service’s environmental footprints at all steps of its life cycle. It includes the following four steps as per ISO 2006 standards.

#### 2.5.1. Goal and scope definition

The purpose of an LCA-based study should be mentioned before the research begins, and it should clearly define the study’s objective as well as the target audience for whom the data will be presented. The goal of the LCA research has repercussions for all other parts of the study, including the technique and level of detail used in LCI, impact assessment analysis, and reporting. The study’s goal was to do the life cycle assessment of biomass pellets from various agroforestry species, as well as to identify the environmental impacts of the biomass pellets, which included pellets of *Cedrus deodar* sawdust, blue pine sawdust, wheat straw, and corn cob, and to estimate cumulative energy demand, materials flows, emissions to water, air, and soil caused by biomass pellet production chain at Lab-scale.

#### 2.5.2. Functional unit

The functional unit should be in line with the study’s goals. One of the essential roles of a functional unit is to act as a point of reference for data inputs and output. This reference is to ensure that LCA findings are comparable, which is especially critical when evaluating many systems, so such comparisons may be made consistently. One kilogram of biomass pellets manufactured from agricultural and forestry wastes served as a functional unit in the present study.

#### 2.5.3. System boundary of the study. Fig 2

#### 2.5.4. Life-cycle inventory

LCI assesses the emissions of the inputs and output flows. Materials and energy are included on the input side. The output side contains all products, by-products, and wastes released into the environment (air, water, and land). For creating a flow model system using the output and input data. The flow model displays the many stages that are assessed. The flow chart (Fig 2) illustrates all of the steps that will take place and an apparent explanation of the boundary system. Only activities within the system boundaries are well thought-out to collect inputs and output data. All facts must be linked to the functional unit, as the aim and scope description specified. This study performed a life cycle inventory for one kg of biomass pellets biofuel produced. The inputs of two softwood tree species sawdust and two agriculture crops residues were used for biomass pellets biofuel production. The output of each selected sample is one kg of pellets production. In *Cedrus deodara* sawdust pellets production 1000-gram (g) of sawdust was mixed with 160 g bio-binder, 120 milliliters (ml) water, 130 g lubricating oil, and consumed 0.0065 kWh electricity. In blue pine sawdust pellets biofuel production, 1000g of sawdust mixed with the materials of; 150 g bio-binder, 120 ml water, 130 g lubricating oil, and 0.0053 kWh electricity were consumed, respectively. In corncob pellets biofuel production, 1000 g of corncob materials were mixed with the 180 g of bio-binder,140 g lubricating oil, and 130 ml water and 0.0067 kWh electricity used, respectively. Around 1000 g wheat straw, 200 g bio-binder,150 ml water, 140 g lubricating oil, and 0.0055 kWh electricity were used for wheat straw pellets biofuel production, as shown in Table 1.

**Table 1:** LCI for biomass pellets biofuel production from agroforest residues in Gilgit-Baltistan, Pakistan.

**Inputs**	**unit**	**Deodar sawdust**	**Blue pine**	**Corncob**	**Wheat straw**
Sawdust	g	1000	1000	1000	1000
Bio-binder	g	160	150	180	200
Lubricating oil use	g	130	130	140	140
Water	mL	130	120	130	150
Electricity consumed	kWh	0.0065	0.0053	0.0067	0.0055
**Output**					
Biomass pellets biofuel	(kg)	1	1	1	1

#### 2.5.5. Life Cycle Impact Assessment (LCIA)

Life Cycle Impact Assessment (LCIA) aims to investigate and transform LCI data into potential ecological impacts. The environmental burdens of biomass pellets were assessed using the CML v2000 methodology present in the SimaPro software v 9.1 software. The life cycle inventory was transformed into environmental impacts following ISO 14040–14044 standard [29].

## 3. Biomass pellets biofuel samples characterization

The manufactured biomass pellets were characterized for investigating their different properties. Characterization of pellets was performed on an experimental basis in the Lab for the following properties of the pellet’s biofuel;

### 3.1. Determination of percent moisture content

**Fig 3:**
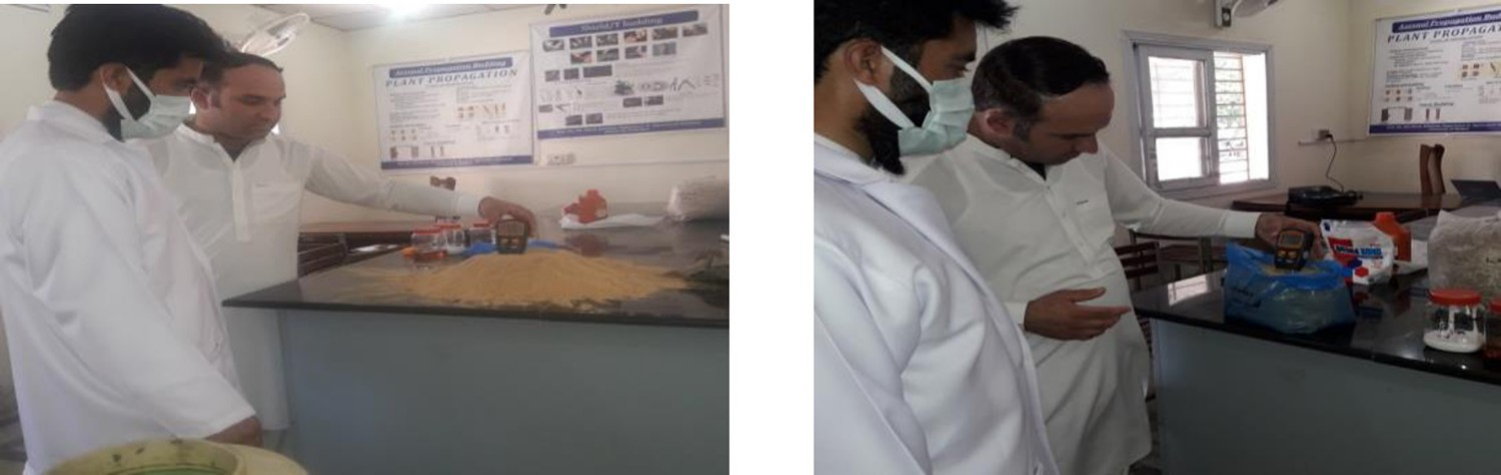
Calculation of moisture content of biomass pellets biofuel prototypes.

Moisture percentage was determined using a laboratory oven and precision balance following the standard of (ASTM D1762). The moisture content is also determined by using a moisture tester as can be seen in Fig 3. For pellets biofuel produced in the present study, the percentage of the moisture content of samples was (<15%).

The percentage of moisture content was determined by using the following Eq # 1;

Moisture(%)=[(A‐B)/A]+100 (1)

Where,

A = Air dried sample, B = Oven dried sample at 105C⁰±1

### 3.2. Calculation of pellet dimension

**Fig 4:**
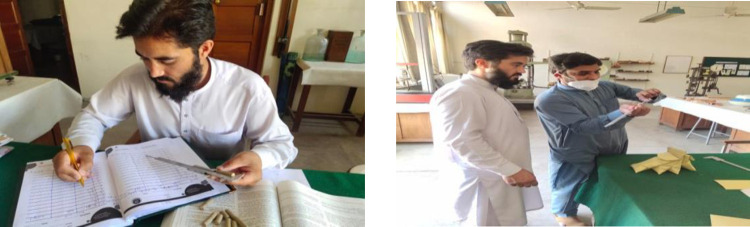
Measuring of length and diameter of biomass pellets.

The pellets’ dimensions were determined using instruments called vernier caliper and scale. Randomly 20 pellets were selected from each sample to determine the average length and diameter, each pellet has an equal diameter, but different lengths and all the pellets are cylindrical (Fig 4).

### 3.3. Calculation of pellets ash content

**Fig 5:**
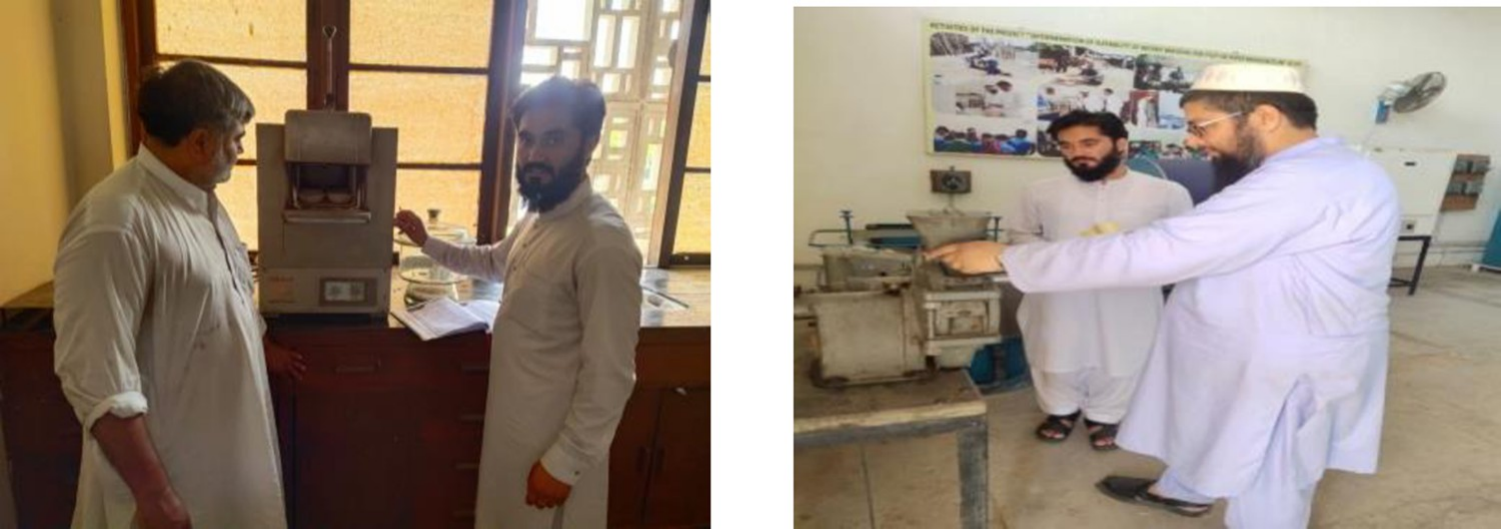
Calculation of ash content for biomass pellets biofuel.

The procedure for measuring the ash content of biomass pellets is;

The first step is to crush the biomass pellets using a machine Willy mill, and 20-gram samples are obtained. For preparing 20gram samples, a Willy mill machine takes five minutes for each sample. These samples are oven-dried at 105C⁰±1 for 45 minutes The oven-dried weight of each sample is noted. Afterward noted oven-dried weight, we found the gram of residues; the samples were placed in the muffle furnace through the porcelain crucible for 25 minutes at a temperature of 575C⁰±5C⁰ to the standard of (TAPPI T211 om-85) (Fig 5). After 25 minutes, the samples were extracted from the muffle furnace, and these samples were cooled for 45 minutes; then, the sample’s residues and obtained weights of residues were put to the Eq # 2, as given below.


AshContent(%)=(D/Wo)×100 (2)

Where, D = oven-dried weight of the sample, wo = Grams of residues

### 3.4. Bulk density

The bulk density was calculated using Eq # 4; the oven-dried weight of samples divided by sample volume. Volume can be calculated through Formula # 3;


$$V=L\times \pi r^{2}$$ (3)

Where L = average length of sample in mm, π = 22/7, r (radius) = d/2, d = average diameter of samples in mm.

The diameter and length of biomass pellets were measured by scale and vernier caliper.


ρ=wo/v (4)

Where, ρ = bulk density (g/cm^3^), wo = oven dry weight in g and v = volume of pellet in cm^3^

### 3.5. Calorific value

The biomass pellet’s calorific value was determined by using two different formulas;

**Delong’s formula**:

HHV=4.18×(78.4×C+241.3×H+22.1×S) (5)

Where C represents carbon in %, H represents hydrogen in %, and S represents sulphur in %.

**Vandralek formula**:

HHV=4.18×[85×C+270×H+26×(S‐O)] (6)

C represents carbon in %, H represents hydrogen in %, and S represents sulphur in %. Eq 5 was used for HHV calculation in the present study.

Low heating value (LHV) can be obtained through the following formula.


LHV=HHV–2.447{H%/100}9.001 (7)

And, another direct formula is used for measuring low heating value, i.e.


LHV=4.18×(94.14×C‐0.5501‐52.14×H) (8)

Eq 8 was used for LHV calculation in the present study.

### 3.6. Elemental analysis

Major elements were investigated in biomass pellets through elemental analysis. The values of major elements like C, H, O, and S were measured through SEM (Scanning Electron Microscope JSM-IT100) in the National Center of Excellence in Geology, University of Peshawar, Pakistan.

## 4. Results and discussion

### 4.1. Biomass pellets biofuel characterizations results

In our study, biomass pellets are manufactured from two agricultural crop residues, such as wheat straw and corncob, and two species of softwood trees, such as deodar and blue pine trees.

The diameter (8.17mm) of each four species of pellets was the same due to the regular hole size in the ring die of the pelletizer machine as shown in Table 2. Table 2 shows that the length of biomass pellets varies, with wheat straw pellets having the most extended length (48mm), followed by blue pine sawdust (29.5mm), deodar sawdust (32.13mm), and corncob (43.46mm). when compared to the Italian standard length(≤38mm) of wood pellets, our pellet’s length was average within the standard-length size. According to various previously published research studies, the moisture content percentage of biomass pellets is 15%. Before pelletizing, the moisture percentage of our pellet’s samples (agriculture crops and forest trees leftovers) is less than 5%. Some water is added to increase the moisture content required to improve pellet quality during pellets production. After biomass pellets production from agroforestry residues, the moisture content is increased due to adhesives by 15%, 16%, 16.5%, and 17% for deodar sawdust, blue pine sawdust corncob, and wheat straw pellets. When pellets are oven-dried according to ASTM D1762-28, the pellet’s moisture content is shown in Table 2, such as deodar sawdust pellets’ moisture content of 5.93%, blue pine pellets’ moisture content of 5.2%, wheat straw pellets’ 11.91%, and corncob pellets’ 5.09%. While comparing to the recommended moisture content (≤10), the moisture content of our biomass pellets was lower than 10%, as demonstrated in Table 2. The higher the moisture percentage in biomass samples significantly influences the length and durability of the production of biomass pellets. The highest ash content of biomass pellets was reported for wheat straw pellets with 1.68%, followed by deodar sawdust at 0.76% and corncob pellets at 0.72%, while blue pine sawdust pellets had the lowest ash content at 0.55%. The Italian recommendation for ash content was (≤0.7%). According to our findings, the ash content was within the recommended range as shown in Table 2. Our biomass pellets bulk density results were 462.97, 754.58, 522, and 460.75 kg/m^3^ for deodar, blue pine, wheat straw, and corncob pellets, respectively, as shown in Table 2. The bulk density of blue pine sawdust is slightly greater than the Italian recommended range and other three species pellets such as; deodar, wheat straw, and corncob pellets ranges are lower than the recommended Italian standard, as the Italian standard recommended range was 620–720 kg/m^3^. Biomass pellets are manufactured from agro-forestry residues such as deodar sawdust, blue pine sawdust, corncobs, and wheat straw. After the pellets production, we used the Dulong’s formula for the calculation of the calorific value, and the calorific values of our selected studied biomass pellets were 22.42 MJ/kg, 23.76 MJ/kg, 22.19 MJ/kg, and 21.14 MJ/kg for deodar, blue pine, wheat straw, and corncob, respectively. Blue pine sawdust pellets have the highest heating value compared to the other three biomass pellets, as shown in Table 2. All the selected biomass pellets’ calorific values, such as high heating values, are the same as the Italian recommended CTI-R04/05 value (>16.91MJ/kg). The Blue pine sawdust pellets have the highest low heating value of 22 MJ/kg as compared to the other three species’ pellets’ values of 20.42, 20.13, and 18 MJ/kg for Deodar sawdust pellets, wheat straw, and corncob pellets, respectively, as summarized in Table 2. While we have calculated low heating values of our developed biomass pellets fall within the recommended range. After elemental analysis of biomass pellets through a scanning electron microscope, the values of nitrogen obtained were: 0.62%, 0.82%, 0.71%, and 0.62% for deodar sawdust pellets, blue pine, wheat straw, and corncob pellets, respectively. The sulfur percentage in our manufactured biomass pellets and 0.82% and 0.91% in blue pine and corncob, while the two species of pellets, such as deodar pellets and wheat straw pellets, have sulfur percentages not detected (ND).

**Table 2:** Characterization of biomass pellets from different agro-forestry species residues.

Pellets Parameters	Unit	Deodar tree	Blue pine tree	Wheat straw	Corncob	Italian standard
Diameter	mm	8.17	8.17	8.17	8.17	6±0.5–8
Length	mm	32.13	29.5	48	43.46	>3.8
Moisture content	%	5.93	5.2	6	5.8	≤10
Ash content	%	0.76	0.55	1.68	0.72	≤0.7
Bulk density	kg/m^3^	462.97	754.58	522	460.75	620–720
Nitrogen	%	0.62	0.82	0.71	0.74	≤0.3
Sulphur	%	ND	0.82	ND	0.91	≤0.5
High heating values	MJ/kg	22.41	23.76	22.19	22.14	>16.91
Low heating values	MJ/kg	20	22	20	18	>1.77

### 4.2. Life cycle impact assessment

There are many environmental impact categories caused by the production processes of the biomass pellets biofuel such as acidification (0.006 kg SO_2_ eq/kg pellets), abiotic depletion (0.018 kg Sb eq/kg pellets), marine aquatic ecotoxicity (417.803 kg 1,4-DB eq/kg pellets), human toxicity (1.107 kg 1,4-DB eq/kg pellets), freshwater aquatic ecotoxicity (0.191 kg 1,4-DB eq/kg pellets), eutrophication (0.001 kg PO_4_ eq/kg pellets), global warming (0.802 kg CO_2_ eq/ kg pellets), and terrestrial ecotoxicity (0.008 kg 1,4-DB eq/kg pellets) respectively as shown in Table 3. The results showed that wheat straw pellets have the highest contribution to the following impact categories: acidification, abiotic depletion, marine aquatic ecotoxicity, freshwater aquatic ecotoxicity, eutrophication, global warming, and terrestrial ecotoxicity, respectively. In contrast, the blue pine pellets contribute less to the above-mentioned environmental impact categories during the biomass pellets production in Gilgit Baltistan, as shown in Table 3. The highest environmental impacts were from fossil fuels i.e., lubricating oil used during biomass pellets production. Moreover, the emissions of various contaminants/pollutants to different environmental compartments such as air, soil, and water are summarized in Supplementary Information-contains all the Tables from Tables A1 to A10 in S1 File.

**Table 3:** Environmental impacts posed by biomass pellets production from selected agro-forest species sawdust/residues.

Impact category	Unit	Blue pine pellets	Deodar pellets	Wheat straw pellets	Corncob Pellets
Abiotic depletion	kg Sb eq	0.005	0.005	0.006	0.002
Acidification	kg SO_2_ eq	0.001	0.001	0.002	0.002
Global Warming Potential	kg CO_2_ eq	0.172	0.175	0.266	0.189
Human toxicity	kg 1,4-DB eq	0.119	0.119	0.74	0.129
Freshwater ecotoxicity	kg 1,4-DB eq	0.04	0.041	0.066	0.044
Marine aquatic ecotoxicity	kg 1,4-DB eq	94.179	94.666	127.1107	101.841
Terrestrial ecotoxicity	kg 1,4-DB eq	0.001	0.001	0.005	0.001

### 4.3. Cumulative energy demand

**Fig 6:**
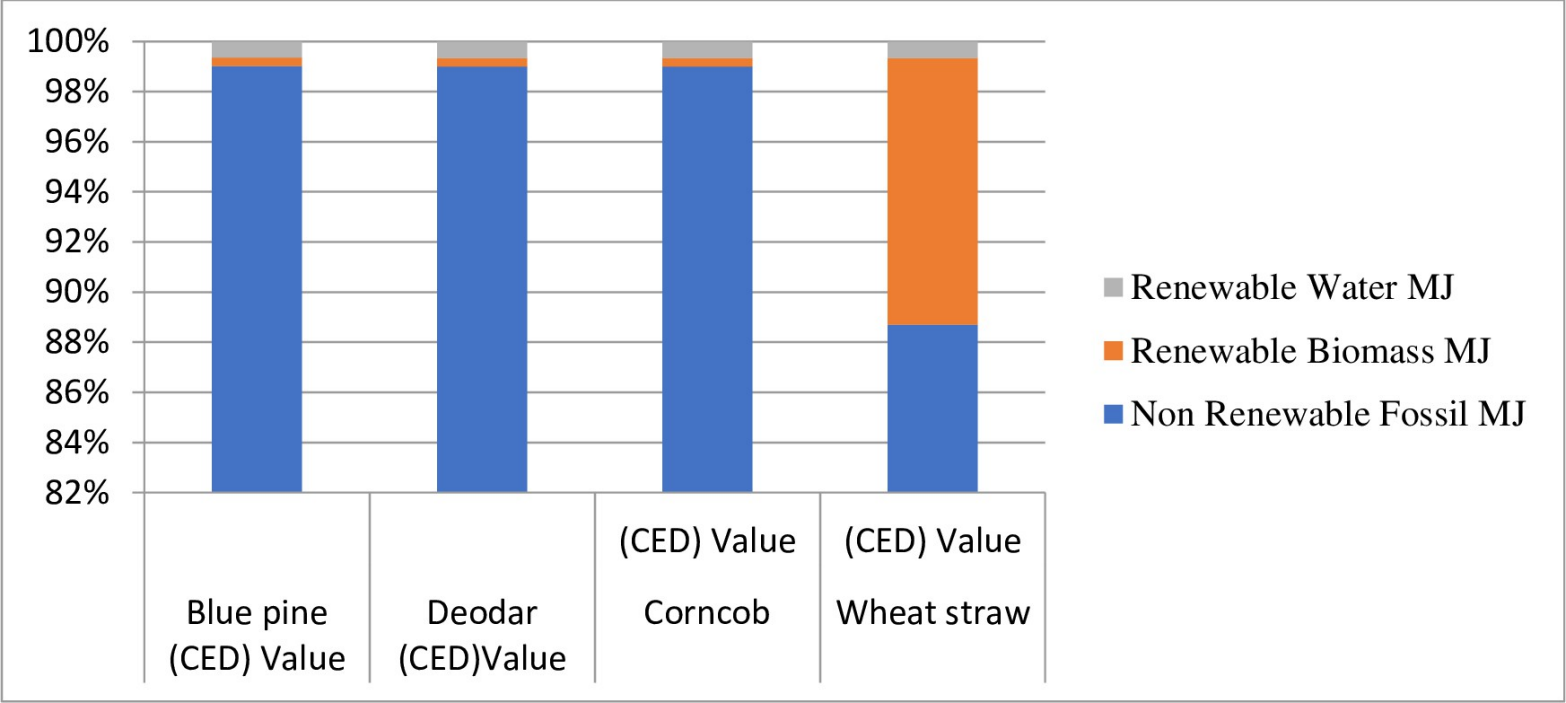
Cumulative energy demand (CED) of biomass pellets biofuel.

The cumulative energy demand of biomass pellets from selected agroforestry species was different. Our study results showed that the maximum energy was from wheat straw pellets (13.737 MJ), followed by corncob pellets (11.754 MJ), and deodar sawdust pellets (10.905 MJ), and blue pine sawdust pellets (10.877MJ), as presented in Table 4 and Fig 6. In biomass pellet production, the maximum energy was obtained from non-renewable fossil fuels, such as the pellets of wheat straw (11.669 MJ), followed by corncob (11.639 MJ), deodar sawdust (10.796 MJ), and lowest in blue pine pellets (10.771 MJ), respectively. In biomass pellet production processes, the contribution of input renewable biomass in blue pine sawdust and deodar sawdust pellets were the same, 0.037 and 0.037 MJ, and the other two agro-crop pellets, such as corncob and wheat straw, values are different with each other, 0.039 and 1.465 MJ. The lowest energy obtained during the biomass pellets production was renewable water such as blue pine sawdust and proceeded deodar sawdust, corncob, and wheat straw have the highest values of 0.069, 0.072, 0.077, and 0.090 MJ as shown in Table 4 and Fig 6. The energy obtained from the selected non-renewable biomass input is negligible and significantly less contributed to wheat straw pellets.

**Table 4:** Cumulative energy demand of biomass pellets from different agroforestry species residues.

Impact category	Unit	Blue pine CED	Deodar CED	Corncob CED	Wheat straw CED
Non-Renewable Fossil	MJ	10.771	10.796	11.638	12.217
Renewable Biomass	MJ	0.037	0.037	0.039	1.465
Renewable Water	MJ	0.069	0.072	0.077	0.090
**Total CED**	**MJ**	**10.877**	**10.905**	**11.754**	**13.737**

### 4.4. Water footprint of biomass pellets biofuel from different agro-forest species residues

**Fig 7:**
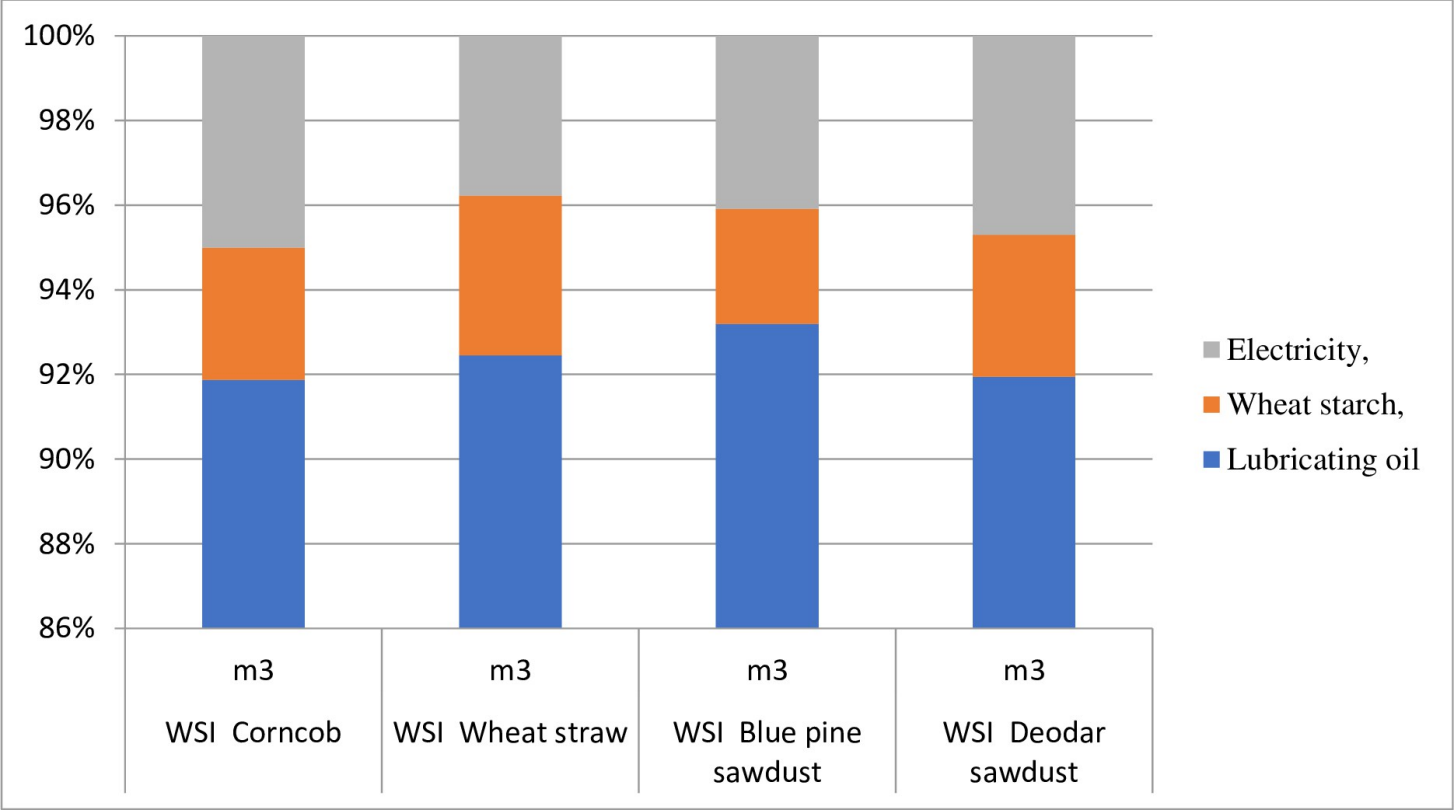
Water footprints of biomass pellets from various agroforest species residues.

The water footprint (WSI) or scarcity index of water for one kg of biomass pellets production in Gilgit-Baltistan, Pakistan during 2020–21 is summarized in Table 5. The water footprint for the production of one kg of corncob pellets was calculated as 0.00160 m^3^. Among the various production activities, collection and transportation of primary raw material, crushing, screening, adding adhesives, pelletizing, cooling, final screening, and packing have the maximum contribution to the water scarcity index, followed by lubricating oil (0.00147m^3^). In contrast, the minimum contribution to water footprint was from electricity (0.00008m^3^) and wheat starch (0.00005m^3^), as shown in Table 5 and Fig 7. Our study results showed that the water footprint for manufacturing one kg of Wheat straw pellets was calculated as (0.03938 m^3^). The following inputs were used; lubricating oil, wheat starch, and electricity. The highest contribution to the water scarcity index was from lubricating oil followed by wheat straw and electricity 0.03938, 0.00006, and 0.00006 m^3^ as demonstrated in Table 5 and Fig 7. Water footprint for the production of one kg softwood tree species i.e., blue pine sawdust pellets was calculated as 0.00147 m^3^. The maximum contribution to the water scarcity index was from lubricating oil (0.00147 m^3^). The results of our study exhibited that the water scarcity index for one kg of Deodar tree sawdust pellets was 0.00149 m^3^.

**Table 5:** Water footprint of biomass pellets from various agro-forest species residues.

Impact category	Unit	Lubricating oil	Wheat starch,	Electricity,	Total
WSI Corncob	m^3^	0.00147	0.00005	0.00008	0.00160
WSI Wheat straw	m^3^	0.00147	0.00006	0.00006	0.03938
WSI Blue pine sawdust	m^3^	0.00137	0.00004	0.00006	0.00147
WSI Deodar sawdust	m^3^	0.00137	0.00005	0.00007	0.00149

### 4.5. Ecological footprint of biomass pellets biofuel from selected agricultural and forestry species residues

The ecological footprint results for one kg of biomass pellets production and its relative contribution per process in districts Diamer, Astore, and Gilgit in Gilgit-Baltistan, Pakistan during 2020–21 are shown in Table 6. Our study results showed that the total ecological footprint for impact categories such as carbon dioxide, nuclear, and land occupation for producing one kg blue pine sawdust pellet was calculated as 0.4134, 0.0321, and 0.0458 m^2^a. The highest contribution to the ecological footprint impact categories such as carbon dioxide, nuclear, and land occupation was lubricating oil and less contribution of wheat starch and electricity for manufacturing one kg blue pine sawdust pellets as demonstrated in Table 6. The results showed that lubricating oil has the highest contribution to the production of Deodar sawdust pellets in ecological footprint impact categories was calculated such as carbon dioxide (0.3531m^2^a), nuclear (0.0320 m^2^a), and land occupation (0.0119 m^2^a). The other two inputs, wheat straw, and electricity had a minor contribution to ecological footprint impact categories as shown in Table 6. The overall environmental impact categories were calculated, such as carbon dioxide (0.4184 m^2^a), nuclear (0.0321 m^2^a), and land occupation (0.0481 m^2^a) for the manufacturing of one kg deodar sawdust pellets, as shown in Table 6. The manufacturing of one kg Corncob pellet’s total ecological footprint impact categories was calculated such as carbon dioxide (0.4530 m^2^a), nuclear (0.0346 m^2^a), and land occupation (0.0535 m^2^a), as shown in Table 6. Ecological footprint impact categories were calculated for producing 1 kg wheat straw pellets. Our study results showed that the environmental footprint for carbon dioxide, nuclear, and land occupation was 0.5565, 0.0429, and 0.4936 m^2^a, as shown in Table 6.

**Table 6:** Ecological footprint of biomass pellets biofuel from Agro-forestry species residues.

Impact category	Unit	Lubricating oil	Wheat starch,	Electricity,	Total
Carbon dioxide blue pine tree	m^2^a	0.3531	0.0539	0.0063	0.4134
Carbon dioxide Deodar tree	m^2^a	0.3531	0.0575	0.0077	0.4184
Carbon dioxide Corncob	m^2^a	0.3803	0.0647	0.0080	0.4530
carbon dioxide Wheat straw	m^2^a	0.3803	0.0719	0.0065	0.5565
Nuclear Blue pine tree	m^2^a	0.0320	-	0.0001	0.0321
Nuclear Deodar tree	m^2^a	0.0320	-	0.0001	0.0321
Nuclear Corncob	m^2^a	0.0345	-	0.0001	0.0346
Nuclear Wheat straw	m^2^a	0.0345	-	0.0001	0.0429
Land occupation blue pine tree	m^2^a	0.0119	0.0338	0.0001	0.0458
Land occupation Deodar tree	m^2^a	0.0119	0.0360	0.0001	0.0481
Land occupation Corncob	m^2^a	0.0128	0.0405	0.0001	0.0535
Land occupation Wheat straw	m^2^a	0.0128	0.0450	0.0001	0.4936

## 5. Discussion

The moisture content percentages for the pellet’s biofuel derived from agro-forest species residues ranged from 5.58 to 6.77 percent in the present study, which was less than 10 percent of the ISO-17225-2 required limit [34]. The present study also determined the moisture content of the biomass pellets biofuel produced from agroforestry residues such as blue pine sawdust, deodar sawdust, wheat straw, and corncob following the Italian recommended standard; there is no significant variation found in moisture content in the present study as shown in Table 2. High moisture percentage level (more than 10%) influences biomass materials’ ignition quality and lowers the power and durability of agro-forest biomass pellets [35]. Analyzing several types of pellets from various sources, we found out that higher bulk density value (over 780 kg/m^3^) did not correspond to high durability levels for pellets biofuel. Pellets made from herbaceous and shrubs biomass and barley and wheat straw have the same problems (with values around 620 kg/m^3^ [36]. The following parameters were determined for pellets made in Finland: moisture content, length, diameter, ash content, net calorific value, and bulk density: typically, 8-millimeter in diameter with 11–30 mm lengths, 7–12 percent, 0.5 percent, 650–700 kg/m^3^, and 4.7–5.0 kWh/kg (16.8–18 MJ/kg). As a result, the energy content of the pellets was estimated to be 3000–3300 kWh/m^3^ or 300–330 L of fuel oil light. Many pellets require approximately a 1.5-meter cube of handling space and are equivalent to 470–500 liters of glow fuel oil. Wood pellets swell and disintegrate when exposed to water; therefore, direct contact with moisture negatively influences [37]. Another study looked at the combustion behavior of four different types of wood pellets and reported that all samples had moisture levels below the European guidelines. Bulk densities likewise surpassed the lower permissible values, and ash composition was identical throughout the four pellet categories studied [38]. It was discovered that ash, sulfur, chlorine, and nitrogen content had more changeability than other parameters. There were no considerable calorific value and nitrogen content changes between quality classes. Consequently, rather than adjusting different parameters, it was suggested that further improving the value of the pellets may be attained by lowering the ash content limit [39]. In the present study, dimensions of our selected agroforestry species pellets such as deodar sawdust pellets, blue pine sawdust, corncob, and wheat straw pellets have an equal diameter of 8.17 mm, there is no significant variation found and comparable to the Italian standard and previous studies results. The biomass pellets biofuel production from agro-forestry selected species have different average lengths such as deodar (32.13 mm), blue pine (29.5 mm), corncob (43.46 mm), and wheat straw (48 mm), as shown in Table 2. In contrast, the length of the species’ pellets followed the previous studies and Italian recommended standards. In this study, pellets made from biomass residues had a relatively high ash content; however, pellets made from sugarcane, bagasse, corncob, and pili had an ash content of 3.0–4.8 percent. The ash percentage of rice straw, tea leaves, tobacco shoots, and banana peduncles ranged from 13.4 to 21.3 percent. Feedstock with a high ash percentage (>0.5%) is often tricky during thermal conversion due to issues with ash removal, slagging, equipment corrosion, and deposit development in the furnace [40].

The high heating value ranged from 18.85 to 22.32 MJ/kg, similar to the 17.78–20.51 MJ/kg reported for oil palm frond pellet [40, 41]. This study shows that the high heating values for biomass pellets were no significant variation among these values and other research values and standard Italian ranges. The life cycle impact assessment of biomass pellets biofuel from selected species, while many environmental impact categories are caused during the biomass pellets’ production processes. To measure biomass pellets’ cumulative energy from different agroforestry species residues. Our study results showed that the maximum cumulative energy was from wheat straw pellets (13.737 MJ), followed by corncob pellets (11.754 MJ), and deodar sawdust pellets (10.905 MJ), and blue pine sawdust pellets (10.877 MJ). The various production operations; are collection and transportation of primary raw material, crushing, screening, adding adhesives, pelletizing, cooling, final screening, and packing [42]. The study investigated the water footprint of biomass pellets. The total water footprint for one kg biomass pellets as; Corncob, Wheat straw, blue pine, and Deodar pellets was calculated as, 0.00160 m^3^, 0.03938 m^3^, 0.00147 m^3,^ and 0.00149 m^3^, respectively.

## 6. Conclusion and recommendations

### 6.1. Conclusion

The present study developed biomass pellets biofuel prototypes from sawdust, bio-binder, lubricating oil, and water. Laboratory tests were applied to characterize the developed biomass pellets biofuel. Biomass pellets from sawdust of blue pine, deodar, corncob, and wheat straw exhibited the same characteristics specified in the Italian standard CTIR 04/5. The moisture content in all pellets produced from two softwood tree species’ sawdust and two agricultural crop species residues was within the range, i.e., less than 10%. The ash content and size of biomass pellets biofuel from agricultural and forestry species were within the acceptable limit. The length of biomass pellets is primarily in correlation to moisture content. The length of agro-forestry species pellets decreases as moisture content increases. Similarly, when bulk density was measured, blue pine sawdust pellets had the highest bulk density compared to other chosen species of pellets. High heating values (HHV) and low heating values (LHV) were computed. Blue pine sawdust pellets had the highest heating value compared to biomass pellets from the other three species. However, heating values were generally within the range and more significant than the suggested Italian standard. Sulfur and nitrogen content were also measured, and the values were within the acceptable ranges, but nitrogen levels were more significant in deodar and wheat straw pellets. The highest contribution to water footprint is wheat straw pellets, and the other three agroforestry species, such as deodar, blue pine, and corncob pellets, have much less contributed. The ecological footprint of biomass pellets from selected agroforestry species residues was calculated. Our study results show that the total ecological footprint for impact categories such as carbon dioxide, nuclear, and land occupation for the production of one kg of agriculture and forestry species pellets was calculated; every species pellet has different ecological footprints.

### 6.2. Recommendations

Eco-industrial park (EIP) concept should be introduced in which waste materials from sawmills and plywood industries should be used as a raw material for biomass pellets biofuel production. Biomass and biofuel are emerging fields, and government and higher authorities should focus on research in this field. In Pakistan, the biomass pellets industry is one of the emerging fields. In other American and European countries, they are already using wood pellets to decrease their dependency on fossil fuels. In the pellets production in the pelletizer machine, no or very little lubricating oil should be used as they are the hotspot source for environmental burdens. Pakistan’s newly established province, Gilgit-Baltistan, has no gas pipeline. Peoples use wood for heating and cooking purposes; therefore, the people are illicitly cutting trees, and due to cutting of trees, deforestation causes which ultimately causes climate change. Consequently, it is strongly recommended that the Government of Gilgit-Baltistan should introduce the renewable biomass pellets biofuel industry in the Gilgit-Baltistan province of Pakistan to mitigate the perilous impacts of deforestation and climate change.

## Supporting information

S1 File (DOCX)
