# Supplementary material for: Life cycle environmental sustainability and cumulative energy assessment of biomass pellets biofuel derived from agroforest residues
Source: PLoS One. 2022 Oct 7;17(10):e0275005. doi: 10.1371/journal.pone.0275005 (PMC9543634; doi:10.1371/journal.pone.0275005)
Supplement: S1 File — (DOCX) [file pone.0275005.s001.docx]

**APPENDICES**

**Table A1**

| **Emissions of various pollutants to water from Blue pine biomass pellet production** | | | | | | | | | | | | | | | | | | | | |
| --- | --- | --- | --- | --- | --- | --- | --- | --- | --- | --- | --- | --- | --- | --- | --- | --- | --- | --- | --- | --- |
| **Substances** | | | | | | | **Unit** | | | | **Total** | | | | | | |  |  |  |
| Antimony-124 | mBq | | | | | | | | | | 13.983 | | | | | | |  |  |  |
| Antimony-125 | µBq | | | | | | | | | | 257.699 | | | | | | |  |  |  |
| Adsorbable Organic Halogen as Cl | µg | | | | | | | | | | 25.339 | | | | | | |  |  |  |
| Arsenic | µg | | | | | | | | | | 388.237 | | | | | | |  |  |  |
| Arsenic V | Ng | | | | | | | | | | 13.698 | | | | | | |  |  |  |
| Asbestos | Pg | | | | | | | | | | 0.003 | | | | | | |  |  |  |
| Asulam | Ng | | | | | | | | | | 2.192 | | | | | | |  |  |  |
| Atrazine | Pg | | | | | | | | | | 542.66 | | | | | | |  |  |  |
| Azadirachtin | Ng | | | | | | | | | | 67.991 | | | | | | |  |  |  |
| Azoxystrobin | µg | | | | | | | | | | 1.43 | | | | | | |  |  |  |
| Barite | Mg | | | | | | | | | | 16.001 | | | | | | |  |  |  |
| Barium | Mg | | | | | | | | | | 11.471 | | | | | | |  |  |  |
| Barium-140 | µBq | | | | | | | | | | 11.609 | | | | | | |  |  |  |
| Bensulfuron methyl ester | Ng | | | | | | | | | | 9.999 | | | | | | |  |  |  |
| Bentazone | Ng | | | | | | | | | | 211.266 | | | | | | |  |  |  |
| Benzene | µg | | | | | | | | | | 765.475 | | | | | | |  |  |  |
| COD, Chemical Oxygen Demand | G | | | | | | | | | | 2.207 | | | | | | |  |  |  |
| Copper | Mg | | | | | | | | | | 3.636 | | | | | | |  |  |  |
| Cresol | Pg | | | | | | | | | | 16.811 | | | | | | |  |  |  |
| Cu-HDO | Pg | | | | | | | | | | 1.276 | | | | | | |  |  |  |
| Cumene | µg | | | | | | | | | | 41.664 | | | | | | |  |  |  |
| Curium alpha | µBq | | | | | | | | | | 56.713 | | | | | | |  |  |  |
| Dibenz (a,h)anthracene | Pg | | | | | | | | | | 0.091 | | | | | | |  |  |  |
| Dibenzofuran | Pg | | | | | | | | | | 0.278 | | | | | | |  |  |  |
| Dibenzothiophene | Pg | | | | | | | | | | 0.23 | | | | | | |  |  |  |
| Dicamba | Ng | | | | | | | | | | 189.902 | | | | | | |  |  |  |
| Ethene, trichloro- | Pg | | | | | | | | | | 0.001 | | | | | | |  |  |  |
| Ethephon | Ng | | | | | | | | | | 535.313 | | | | | | |  |  |  |
| Ethyl acetate | Ng | | | | | | | | | | 1.894 | | | | | | |  |  |  |
| Fluoride | Mg | | | | | | | | | | 17.612 | | | | | | |  |  |  |
| Fluorine | Ng | | | | | | | | | | 24.304 | | | | | | |  |  |  |
| Fluorodifen | Ng | | | | | | | | | | 16.874 | | | | | | |  |  |  |
| Fluosilicic acid | µg | | | | | | | | | | 4.83 | | | | | | |  |  |  |
| Fluroxypyr | Ng | | | | | | | | | | 626.56 | | | | | | |  |  |  |
| **Table A2**  **Emissions of various pollutants to Soil from Blue pine biomass pellet production** | | | | | | | | | | | | | | | | | | | | |
| **Substances** | | | | | | | **Unit** | **Total** | | | | | | | | | | | | |
| Ametryn | | | | | | | Ng | 2.175 | | | | | | | | | | | | |
| Amidosulfuron | | | | | | | Ng | 899.952 | | | | | | | | | | | | |
| Ammonia | | | | | | | µg | 301.223 | | | | | | | | | | | | |
| Anthraquinone | | | | | | | ng | 1.132 | | | | | | | | | | | | |
| Antimony | | | | | | | ng | 81.192 | | | | | | | | | | | | |
| Barium | | | | | | | mg | 2.634 | | | | | | | | | | | | |
| Benfluralin | | | | | | | ng | 14.737 | | | | | | | | | | | | |
| Benomyl | | | | | | | pg | 418.393 | | | | | | | | | | | | |
| Bensulfuron methyl ester | | | | | | | ng | 899.955 | | | | | | | | | | | | |
| Bentazone | | | | | | | µg | 19.014 | | | | | | | | | | | | |
| Benzene, pentachloronitro- | | | | | | | pg | 626.425 | | | | | | | | | | | | |
| Beryllium | | | | | | | pg | 0.002 | | | | | | | | | | | | |
| Bifenox | | | | | | | µg | 11.643 | | | | | | | | | | | | |
| Bifenthrin | | | | | | | ng | 562.692 | | | | | | | | | | | | |
| Cyfluthrin | | | | | | | µg | 13.093 | | | | | | | | | | | | |
| Cyhalothrin, gamma- | | | | | | | pg | 10.008 | | | | | | | | | | | | |
| Cymoxanil | | | | | | | ng | 170.939 | | | | | | | | | | | | |
| Ethalfluralin | | | | | | | pg | 574.871 | | | | | | | | | | | | |
| Fenoxaprop ethyl ester | | | | | | | pg | 147.067 | | | | | | | | | | | | |
| Fenpiclonil | | | | | | | ng | 963.985 | | | | | | | | | | | | |
| Fenpropathrin | | | | | | | pg | 11.506 | | | | | | | | | | | | |
| Fenpropidin | | | | | | | ng | 4.071 | | | | | | | | | | | | |
| Glufosinate | | | | | | | ng | 16.426 | | | | | | | | | | | | |
| Glyphosate | | | | | | | µg | 168.939 | | | | | | | | | | | | |
| Hexazinone | | | | | | | ng | 2.015 | | | | | | | | | | | | |
| Hydramethylnon | | | | | | | pg | 46.434 | | | | | | | | | | | | |
| **Table A3**    **Emissions of various pollutants to Air from Blue pine biomass pellet production** | | | | | | | | | | | | | | |  |  |  |  |  |  |
| **Substances** | | | | **Unit** | | | | | | | | | **Total** | | |  |  |  |  |  |
| Arsenic | | | | µg | | | | | | | | | 45.844 | | |  |  |  |  |  |
| Arsenic trioxide | | | | pg | | | | | | | | | 1.587 | | |  |  |  |  |  |
| Arsenic V | | | | pg | | | | | | | | | 178.000 | | |  |  |  |  |  |
| Arsine | | | | pg | | | | | | | | | 131.746 | | |  |  |  |  |  |
| Asbestos | | | | pg | | | | | | | | | 0.000 | | |  |  |  |  |  |
| Benzene, 1,3,5-trimethyl- | | | | µg | | | | | | | | | 1.602 | | |  |  |  |  |  |
| Benzene, chloro- | | | | pg | | | | | | | | | 0.006 | | |  |  |  |  |  |
| Benzene, ethyl- | | | | µg | | | | | | | | | 286.702 | | |  |  |  |  |  |
| Benzene, hexachloro- | | | | ng | | | | | | | | | 9.710 | | |  |  |  |  |  |
| Benzene, pentachloro- | | | | pg | | | | | | | | | 214.755 | | |  |  |  |  |  |
| Benzo(a)anthracene | | | | pg | | | | | | | | | 169.206 | | |  |  |  |  |  |
| Copper | | | | µg | | | | | | | | | 183.517 | | |  |  |  |  |  |
| Crotonaldehyde | | | | µg | | | | | | | | | 3.745 | | |  |  |  |  |  |
| Cumene | | | | µg | | | | | | | | | 17.350 | | |  |  |  |  |  |
| Dimethoate | | | | µg | | | | | | | | | 2.092 | | |  |  |  |  |  |
| Dimethyl malonate | | | | pg | | | | | | | | | 204.082 | | |  |  |  |  |  |
| Ethanol | | | | µg | | | | | | | | | 45.164 | | |  |  |  |  |  |
| Ethene | | | | µg | | | | | | | | | 740.898 | | |  |  |  |  |  |
| Fenoxaprop-P ethyl ester | | | | ng | | | | | | | | | 202.488 | | |  |  |  |  |  |
| Flumioxazin | | | | pg | | | | | | | | | 346.981 | | |  |  |  |  |  |
| Fluoranthene | | | | ng | | | | | | | | | 1.080 | | |  |  |  |  |  |
| Fluorene | | | | ng | | | | | | | | | 3.548 | | |  |  |  |  |  |
| Glyphosate | | | | µg | | | | | | | | | 16.719 | | |  |  |  |  |  |
| Hexadecane | | | | ng | | | | | | | | | 355.947 | | |  |  |  |  |  |
| Hexamethylene diamine | | | | pg | | | | | | | | | 2.785 | | |  |  |  |  |  |
| Hexane | | | | mg | | | | | | | | | 6.088 | | |  |  |  |  |  |
| **Table A4**  **Emissions of various pollutants to Soil from Deodar biomass pellet production** | | | | | | | | | | | | | | | | | | |  |  |
| **Substances** | | | | | **Unit** | | | | | | | | **Total** | | | | | | |  |
| Aldrin | | | | | µg | | | | | | | | 3.539 | | | | | | |  |
| Alpha-cypermethrin | | | | | ng | | | | | | | | 746.816 | | | | | | |  |
| Aluminum | | | | | mg | | | | | | | | 5.370 | | | | | | |  |
| Americium-241 | | | | | µBq | | | | | | | | 12.525 | | | | | | |  |
| Ametryn | | | | | ng | | | | | | | | 2.175 | | | | | | |  |
| Bifenthrin | | | | | ng | | | | | | | | 600.189 | | | | | | |  |
| Bitertanol | | | | | ng | | | | | | | | 600.073 | | | | | | |  |
| Boron | | | | | µg | | | | | | | | 54.390 | | | | | | |  |
| Curium alpha | | | | | µBq | | | | | | | | 24.857 | | | | | | |  |
| Cycloxydim | | | | | pg | | | | | | | | 595.634 | | | | | | |  |
| Cyfluthrin | | | | | µg | | | | | | | | 13.966 | | | | | | |  |
| Cyhalothrin, gamma- | | | | | pg | | | | | | | | 10.008 | | | | | | |  |
| Diclofop | | | | | ng | | | | | | | | 1.764 | | | | | | |  |
| Diclofop-methyl | | | | | ng | | | | | | | | 1.804 | | | | | | |  |
| Dicrotophos | | | | | ng | | | | | | | | 4.491 | | | | | | |  |
| Difenoconazole | | | | | µg | | | | | | | | 138.304 | | | | | | |  |
| Ethephon | | | | | µg | | | | | | | | 51.409 | | | | | | |  |
| Ethofumesate | | | | | ng | | | | | | | | 20.436 | | | | | | |  |
| Ethoprop | | | | | pg | | | | | | | | 576.998 | | | | | | |  |
| Fenamiphos | | | | | ng | | | | | | | | 12.022 | | | | | | |  |
| Fenpropidin | | | | | ng | | | | | | | | 4.069 | | | | | | |  |
| Fenpropimorph | | | | | µg | | | | | | | | 15.735 | | | | | | |  |
| Glufosinate | | | | | ng | | | | | | | | 16.426 | | | | | | |  |
| Linuron | | | | | µg | | | | | | | | 16.381 | | | | | | |  |
| Lithium | | | | | ng | | | | | | | | 4.720 | | | | | | |  |
| Magnesium | | | | | mg | | | | | | | | 4.572 | | | | | | |  |
| Malathion | | | | | ng | | | | | | | | 8.306 | | | | | | |  |
| Maleic hydrazide | | | | | pg | | | | | | | | 631.105 | | | | | | |  |
| Nitrate | | | | | µg | | | | | | | | 5.439 | | | | | | |  |
| Nitrogen | | | | | µg | | | | | | | | 7.084 | | | | | | |  |
| Norflurazon | | | | | ng | | | | | | | | 2.921 | | | | | | |  |
| Oils, biogenic | | | | | µg | | | | | | | | 58.787 | | | | | | |  |
| Oils, unspecified | | | | | mg | | | | | | | | 707.828 | | | | | | |  |
| Orbencarb | | | | | ng | | | | | | | | 48.356 | | | | | | |  |
| Organic carbon | | | | | ng | | | | | | | | 984.682 | | | | | | |  |
| **Table A5**  **Emissions of various pollutants to Air from Deodar biomass pellet production** | | | | | | | | | | | | | | | | |  |  |  |  |
| **Substances** | | | **Unit** | | | | | | | | | | | **Total** | | |  |  |  |  |
| Arsenic | | | µg | | | | | | | | | | | 45.919 | | |  |  |  |  |
| Arsenic trioxide | | | Pg | | | | | | | | | | | 1.693 | | |  |  |  |  |
| Arsenic V | | | Pg | | | | | | | | | | | 189.867 | | |  |  |  |  |
| Arsine | | | Pg | | | | | | | | | | | 140.528 | | |  |  |  |  |
| Benzaldehyde | | | µg | | | | | | | | | | | 7.717 | | |  |  |  |  |
| Benzene | | | Mg | | | | | | | | | | | 2.040 | | |  |  |  |  |
| Cumene | | | µg | | | | | | | | | | | 17.352 | | |  |  |  |  |
| Curium alpha | | | nBq | | | | | | | | | | | 0.000 | | |  |  |  |  |
| Cyanide | | | µg | | | | | | | | | | | 35.367 | | |  |  |  |  |
| Cyanoacetic acid | | | Pg | | | | | | | | | | | 153.749 | | |  |  |  |  |
| Cyclohexane | | | Ng | | | | | | | | | | | 9.717 | | |  |  |  |  |
| Cyfluthrin | | | µg | | | | | | | | | | | 1.397 | | |  |  |  |  |
| Cyhalothrin, gamma- | | | Pg | | | | | | | | | | | 233.463 | | |  |  |  |  |
| Cymoxanil | | | Ng | | | | | | | | | | | 17.999 | | |  |  |  |  |
| Cypermethrin | | | Ng | | | | | | | | | | | 319.755 | | |  |  |  |  |
| Cyproconazole | | | µg | | | | | | | | | | | 1.148 | | |  |  |  |  |
| Decane | | | µg | | | | | | | | | | | 21.452 | | |  |  |  |  |
| Deltamethrin | | | Ng | | | | | | | | | | | 35.998 | | |  |  |  |  |
| Dibenz(a,h)anthracene | | | Pg | | | | | | | | | | | 99.155 | | |  |  |  |  |
| Dicamba | | | µg | | | | | | | | | | | 1.823 | | |  |  |  |  |
| Dichlorophenol | | | Ng | | | | | | | | | | | 647.963 | | |  |  |  |  |
| Dichlorprop | | | Pg | | | | | | | | | | | 18.844 | | |  |  |  |  |
| Dichlorprop-P | | | µg | | | | | | | | | | | 3.402 | | |  |  |  |  |
| Diethyl ether | | | Pg | | | | | | | | | | | 19.802 | | |  |  |  |  |
| Diethylamine | | | Pg | | | | | | | | | | | 331.242 | | |  |  |  |  |
| Diethylene glycol | | | Pg | | | | | | | | | | | 34.851 | | |  |  |  |  |
| Ethyl acetate | | | µg | | | | | | | | | | | 140.977 | | |  |  |  |  |
| Ethylcellulose | | | Ng | | | | | | | | | | | 285.139 | | |  |  |  |  |
| Ethylamine | | | Ng | | | | | | | | | | | 1.585 | | |  |  |  |  |
| Ethylene diamine | | | Ng | | | | | | | | | | | 2.751 | | |  |  |  |  |
| Ethylene oxide | | | Ng | | | | | | | | | | | 290.314 | | |  |  |  |  |
| Ethyne | | | µg | | | | | | | | | | | 34.035 | | |  |  |  |  |
| Fenbuconazole | | | Ng | | | | | | | | | | | 755.957 | | |  |  |  |  |
| Fenoxaprop | | | Pg | | | | | | | | | | | 159.250 | | |  |  |  |  |
| Fenoxaprop-P ethyl ester | | | Ng | | | | | | | | | | | 215.988 | | |  |  |  |  |
| Glyphosate | | | µg | | | | | | | | | | | 17.816 | | |  |  |  |  |
| Heat, waste | | | kJ | | | | | | | | | | | 74.370 | | |  |  |  |  |
| Helium | | | µg | | | | | | | | | | | 458.236 | | |  |  |  |  |
| Heptane | | | Mg | | | | | | | | | | | 2.752 | | |  |  |  |  |
| Heptenophos | | | Ng | | | | | | | | | | | 311.982 | | |  |  |  |  |
| Hexaconazole | | | Ng | | | | | | | | | | | 365.979 | | |  |  |  |  |
| Hexadecane | | | Ng | | | | | | | | | | | 379.677 | | |  |  |  |  |
| Hexamethylene diamine | | | Pg | | | | | | | | | | | 2.970 | | |  |  |  |  |
| Hexane | | | Mg | | | | | | | | | | | 6.090 | | |  |  |  |  |
| Hydrazine, methyl- | | | Pg | | | | | | | | | | | 0.051 | | |  |  |  |  |
| Hydrocarbons, aromatic | | | µg | | | | | | | | | | | 518.329 | | |  |  |  |  |
| Iron | | | µg | | | | | | | | | | | 512.585 | | |  |  |  |  |
| Isobutane | | | Ng | | | | | | | | | | | 190.043 | | |  |  |  |  |
| Isocyanic acid | | | µg | | | | | | | | | | | 4.331 | | |  |  |  |  |
| Isopentane | | | µg | | | | | | | | | | | 1.329 | | |  |  |  |  |
| **Table A6**  **Emissions of various pollutants to Water from Deodar biomass pellet production** | | | | | | | | | | | | | | | | |  |  |  |  |
| **Substances** | | | | **Unit** | | | | | | | | | | **Total** | | |  |  |  |  |
| Ammonia, as N | | | | pg | | | | | | | | | | 0.013 | | |  |  |  |  |
| Ammonium, ion | | | | mg | | | | | | | | | | 1.187 | | |  |  |  |  |
| Aniline | | | | ng | | | | | | | | | | 1.320 | | |  |  |  |  |
| Anthracene | | | | ng | | | | | | | | | | 5.573 | | |  |  |  |  |
| Benzene, ethyl- | | | | µg | | | | | | | | | | 253.031 | | |  |  |  |  |
| Benzene, pentamethyl- | | | | pg | | | | | | | | | | 0.117 | | |  |  |  |  |
| Benzenes, alkylated, unspecified | | | | pg | | | | | | | | | | 23.848 | | |  |  |  |  |
| Benzo(a)anthracene | | | | ng | | | | | | | | | | 2.610 | | |  |  |  |  |
| Benzo(a)pyrene | | | | pg | | | | | | | | | | 0.951 | | |  |  |  |  |
| Benzo(b)fluoranthene | | | | pg | | | | | | | | | | 0.928 | | |  |  |  |  |
| Cobalt-58 | | | | mBq | | | | | | | | | | 6.637 | | |  |  |  |  |
| Cypermethrin | | | | ng | | | | | | | | | | 35.512 | | |  |  |  |  |
| Cyproconazole | | | | ng | | | | | | | | | | 127.613 | | |  |  |  |  |
| Decane | | | | µg | | | | | | | | | | 24.989 | | |  |  |  |  |
| Deltamethrin | | | | ng | | | | | | | | | | 4.000 | | |  |  |  |  |
| Detergent, oil | | | | ng | | | | | | | | | | 1.520 | | |  |  |  |  |
| Diethylamine | | | | ng | | | | | | | | | | 0.795 | | |  |  |  |  |
| Difenoconazole | | | | µg | | | | | | | | | | 1.536 | | |  |  |  |  |
| Diflufenican | | | | ng | | | | | | | | | | 268.879 | | |  |  |  |  |
| Dimethenamid | | | | pg | | | | | | | | | | 5.603 | | |  |  |  |  |
| Dimethoate | | | | ng | | | | | | | | | | 247.986 | | |  |  |  |  |
| Dimethylamine | | | | ng | | | | | | | | | | 1.787 | | |  |  |  |  |
| Ethene, trichloro- | | | | pg | | | | | | | | | | 0.001 | | |  |  |  |  |
| Ethephon | | | | ng | | | | | | | | | | 571.000 | | |  |  |  |  |
| Ethyl acetate | | | | ng | | | | | | | | | | 1.843 | | |  |  |  |  |
| Ethylamine | | | | ng | | | | | | | | | | 3.804 | | |  |  |  |  |
| Ethylene diamine | | | | ng | | | | | | | | | | 6.653 | | |  |  |  |  |
| Ethylene oxide | | | | ng | | | | | | | | | | 31.568 | | |  |  |  |  |
| Fenbuconazole | | | | ng | | | | | | | | | | 83.995 | | |  |  |  |  |
| Fenoxaprop-P ethyl ester | | | | Ng | | | | | | | | | | 23.999 | | |  |  |  |  |
| Fenpiclonil | | | | Ng | | | | | | | | | | 11.333 | | |  |  |  |  |
| Fenpropimorph | | | | Ng | | | | | | | | | | 174.657 | | |  |  |  |  |
| Fluoranthene | | | | Ng | | | | | | | | | | 44.189 | | |  |  |  |  |
| Fluorene | | | | Ng | | | | | | | | | | 15.148 | | |  |  |  |  |
| Fluorene, 1-methyl- | | | | Pg | | | | | | | | | | 0.178 | | |  |  |  |  |
| Fluorenes, alkylated, unspecified | | | | Pg | | | | | | | | | | 1.382 | | |  |  |  |  |
| Fluoride | | | | Mg | | | | | | | | | | 17.766 | | |  |  |  |  |
| Glutaraldehyde | | | | µg | | | | | | | | | | 1.976 | | |  |  |  |  |
| Glyphosate | | | | µg | | | | | | | | | | 1.952 | | |  |  |  |  |
| Heat, waste | | | | kJ | | | | | | | | | | 6.111 | | |  |  |  |  |
| Heptenophos | | | | Ng | | | | | | | | | | 34.665 | | |  |  |  |  |
| Hexaconazole | | | | Ng | | | | | | | | | | 40.664 | | |  |  |  |  |
| Hexadecane | | | | Pg | | | | | | | | | | 94.220 | | |  |  |  |  |
| Hexane | | | | Pg | | | | | | | | | | 1.999 | | |  |  |  |  |
| Hydrocarbons, unspecified | | | | µg | | | | | | | | | | 389.500 | | |  |  |  |  |
| Hydrogen-3, Tritium | | | | Bq | | | | | | | | | | 206.605 | | |  |  |  |  |
| Hydrogen carbonate | | | | µg | | | | | | | | | | 334.257 | | |  |  |  |  |
| Hydrogen chloride | | | | µg | | | | | | | | | | 97.427 | | |  |  |  |  |
| Isoxaben | | | | Ng | | | | | | | | | | 29.332 | | |  |  |  |  |
| Kresoxim-methyl | | | | Ng | | | | | | | | | | 663.642 | | |  |  |  |  |
| **Table A7**  **Emissions of various pollutants to the soil from Corncob biomass pellet production** | | | | | | | | | | | | | | | | |  |  |  |  |
| **Substances** | | **Unit** | | | | | | | **Total** | | | | | | | |  |  |  |  |
| Abamectin | | Pg | | | | | | | 182.85 | | | | | | | |  |  |  |  |
| Acephate | | Ng | | | | | | | 31.41 | | | | | | | |  |  |  |  |
| Acetamide | | Ng | | | | | | | 4.82 | | | | | | | |  |  |  |  |
| Acetamiprid | | Ng | | | | | | | 8.67 | | | | | | | |  |  |  |  |
| Bromacil | | Ng | | | | | | | 4.68 | | | | | | | |  |  |  |  |
| Bromide | | Ng | | | | | | | 96.27 | | | | | | | |  |  |  |  |
| Bromine | | Ng | | | | | | | 77.94 | | | | | | | |  |  |  |  |
| Bromoxynil | | µg | | | | | | | 32.80 | | | | | | | |  |  |  |  |
| Bromuconazole | | µg | | | | | | | 9.11 | | | | | | | |  |  |  |  |
| Carbon dioxide, to soil or biomass stock | | Mg | | | | | | | 1.08 | | | | | | | |  |  |  |  |
| Carfentrazone-ethyl | | Pg | | | | | | | 13.33 | | | | | | | |  |  |  |  |
| Cesium-137 | | mBq | | | | | | | 6.72 | | | | | | | |  |  |  |  |
| Dinoseb | | Pg | | | | | | | 0.02 | | | | | | | |  |  |  |  |
| Dipropylthiocarbamic acid S-ethyl ester | | Ng | | | | | | | 2.68 | | | | | | | |  |  |  |  |
| Diquat | | µg | | | | | | | 3.72 | | | | | | | |  |  |  |  |
| Dithianone | | Pg | | | | | | | 40.03 | | | | | | | |  |  |  |  |
| Diuron | | Ng | | | | | | | 561.48 | | | | | | | |  |  |  |  |
| Endosulfan | | µg | | | | | | | 44.05 | | | | | | | |  |  |  |  |
| Endothall | | Pg | | | | | | | 21.12 | | | | | | | |  |  |  |  |
| Fenamiphos | | Ng | | | | | | | 12.95 | | | | | | | |  |  |  |  |
| Fenbuconazole | | µg | | | | | | | 8.50 | | | | | | | |  |  |  |  |
| Glufosinate | | Ng | | | | | | | 17.69 | | | | | | | |  |  |  |  |
| Glyphosate | | µg | | | | | | | 202.20 | | | | | | | |  |  |  |  |
| Halosulfuron-methyl | | Pg | | | | | | | 1.90 | | | | | | | |  |  |  |  |
| Heat, waste | | J | | | | | | | 0.00 | | | | | | | |  |  |  |  |
| Heptenophos | | µg | | | | | | | 3.51 | | | | | | | |  |  |  |  |
| Imazaquin | | Ng | | | | | | | 67.50 | | | | | | | |  |  |  |  |
| Imazethapyr | | Pg | | | | | | | 266.33 | | | | | | | |  |  |  |  |
| Imidacloprid | | Ng | | | | | | | 46.79 | | | | | | | |  |  |  |  |
| Indoxacarb | | Ng | | | | | | | 16.15 | | | | | | | |  |  |  |  |
| Insecticides, unspecified | | Ng | | | | | | | 1.80 | | | | | | | |  |  |  |  |
| Iodine-129 | | nBq | | | | | | | 0.44 | | | | | | | |  |  |  |  |
| Iodosulfuron | | Pg | | | | | | | 0.61 | | | | | | | |  |  |  |  |
| Iodosulfuron-methyl-sodium | | Pg | | | | | | | 0.66 | | | | | | | |  |  |  |  |
| Ioxynil | | µg | | | | | | | 8.78 | | | | | | | |  |  |  |  |
| **Table A8**  **Emissions of various pollutants to Water from Corncob biomass pellet production** | | | | | | | | | | | | | | | | | | | |  |
| **Substance** | | | **Unit** | | | | | | | | | **Total** | | | | | | | |  |
| Acenaphthene | | | Ng | | | | | | | | | 94.07 | | | | | | | |  |
| Acenaphthylene | | | Ng | | | | | | | | | 9.47 | | | | | | | |  |
| Acetaldehyde | | | µg | | | | | | | | | 3.77 | | | | | | | |  |
| Acetyl chloride | | | Pg | | | | | | | | | 164.60 | | | | | | | |  |
| Acidity, unspecified | | | µg | | | | | | | | | 5.62 | | | | | | | |  |
| Acids, unspecified | | | Pg | | | | | | | | | 0.03 | | | | | | | |  |
| Acrylate | | | Ng | | | | | | | | | 5.46 | | | | | | | |  |
| Ammonia | | | µg | | | | | | | | | 311.07 | | | | | | | |  |
| Bromine | | | Mg | | | | | | | | | 9.17 | | | | | | | |  |
| Bromoxynil | | | Ng | | | | | | | | | 364.47 | | | | | | | |  |
| Bromuconazole | | | Ng | | | | | | | | | 101.24 | | | | | | | |  |
| Butene | | | µg | | | | | | | | | 1.43 | | | | | | | |  |
| Butyl acetate | | | µg | | | | | | | | | 1.27 | | | | | | | |  |
| Butyrolactone | | | Ng | | | | | | | | | 3.67 | | | | | | | |  |
| Cadmium | | | µg | | | | | | | | | 204.09 | | | | | | | |  |
| Calcium | | | G | | | | | | | | | 1.38 | | | | | | | |  |
| Cerium-144 | | | µBq | | | | | | | | | 2.84 | | | | | | | |  |
| Cesium | | | µg | | | | | | | | | 11.31 | | | | | | | |  |
| Dipropylamine | | | Pg | | | | | | | | | 403.64 | | | | | | | |  |
| Ethene | | | µg | | | | | | | | | 17.09 | | | | | | | |  |
| Ethene, chloro- | | | Ng | | | | | | | | | 144.98 | | | | | | | |  |
| Ethene, tetrachloro- | | | Pg | | | | | | | | | 0.00 | | | | | | | |  |
| Formic acid | | | Pg | | | | | | | | | 111.24 | | | | | | | |  |
| Glutaraldehyde | | | µg | | | | | | | | | 2.13 | | | | | | | |  |
| Glyphosate | | | µg | | | | | | | | | 2.20 | | | | | | | |  |
| Heat, waste | | | kJ | | | | | | | | | 6.79 | | | | | | | |  |
| Heptenophos | | | Ng | | | | | | | | | 39.00 | | | | | | | |  |
| Hexaconazole | | | Ng | | | | | | | | | 45.75 | | | | | | | |  |
| Isoproturon | | | µg | | | | | | | | | 7.78 | | | | | | | |  |
| Isoxaben | | | Ng | | | | | | | | | 33.00 | | | | | | | |  |
| Kresoxim-methyl | | | Ng | | | | | | | | | 746.60 | | | | | | | |  |
| Lactic acid | | | Pg | | | | | | | | | 316.19 | | | | | | | |  |
| Lambda-cyhalothrin | | | Ng | | | | | | | | | 5.85 | | | | | | | |  |
| Metsulfuron-methyl | | | Ng | | | | | | | | | 3.00 | | | | | | | |  |
| Molybdenum | | | µg | | | | | | | | | 323.72 | | | | | | | |  |
| Molybdenum-99 | | | µBq | | | | | | | | | 4.61 | | | | | | | |  |
| N-nonylphenol | | | Ng | | | | | | | | | 225.74 | | | | | | | |  |
| Naphthalene | | | Ng | | | | | | | | | 441.86 | | | | | | | |  |
| Organic carbon | | | µg | | | | | | | | | 1.06 | | | | | | | |  |
| Oxadixyl | | | Ng | | | | | | | | | 4.50 | | | | | | | |  |
| p-Cresol | | | Pg | | | | | | | | | 54.50 | | | | | | | |  |
| Paclobutrazol | | | Ng | | | | | | | | | 13.94 | | | | | | | |  |
| Paraffins | | | µg | | | | | | | | | 1.01 | | | | | | | |  |
| **Table A9**  **Emissions of various pollutants to Air from Corncob biomass pellet production** | | | | | | | | | | | | | | | | | | | | |
| **Substance** | | | | | | **Unit** | | | | **Total** | | | | | | | | | | |
| 1-Butanol | | | | | | pg | | | | 152.29 | | | | | | | | | | |
| 1-Butene | | | | | | µg | | | | 1.94 | | | | | | | | | | |
| 2-Chloroacetophenone | | | | | | pg | | | | 0.00 | | | | | | | | | | |
| 2,4-D amines | | | | | | pg | | | | 14.17 | | | | | | | | | | |
| Fenbuconazole | | | | | | Ng | | | | 850.45 | | | | | | | | | | |
| Fenoxaprop | | | | | | Pg | | | | 171.50 | | | | | | | | | | |
| Fenoxaprop-P ethyl ester | | | | | | Ng | | | | 242.99 | | | | | | | | | | |
| Fluorine | | | | | | µg | | | | 14.85 | | | | | | | | | | |
| Fluorodifen | | | | | | Ng | | | | 182.24 | | | | | | | | | | |
| Fluosilicic acid | | | | | | µg | | | | 2.68 | | | | | | | | | | |
| Fluroxypyr | | | | | | µg | | | | 6.77 | | | | | | | | | | |
| Glyphosate | | | | | | µg | | | | 20.03 | | | | | | | | | | |
| Heat, waste | | | | | | kJ | | | | 83.38 | | | | | | | | | | |
| Helium | | | | | | µg | | | | 493.49 | | | | | | | | | | |
| Heptane | | | | | | Mg | | | | 2.96 | | | | | | | | | | |
| Heptenophos | | | | | | Ng | | | | 350.98 | | | | | | | | | | |
| Hexaconazole | | | | | | Ng | | | | 411.73 | | | | | | | | | | |
| Hexadecane | | | | | | Ng | | | | 427.14 | | | | | | | | | | |
| Hexamethylene diamine | | | | | | Pg | | | | 3.34 | | | | | | | | | | |
| Hexane | | | | | | Mg | | | | 6.56 | | | | | | | | | | |
| Hydrazine, methyl- | | | | | | Pg | | | | 0.06 | | | | | | | | | | |
| Hydrocarbons, aliphatic, alkanes, cyclic | | | | | | µg | | | | 15.57 | | | | | | | | | | |
| Hydrocarbons, aliphatic, alkanes, unspecified | | | | | | µg | | | | 895.87 | | | | | | | | | | |
| Hydrocarbons, aliphatic, unsaturated | | | | | | µg | | | | 164.99 | | | | | | | | | | |
| Ioxynil | | | | | | Ng | | | | 877.45 | | | | | | | | | | |
| Iprodione | | | | | | Ng | | | | 722.21 | | | | | | | | | | |
| Iron | | | | | | µg | | | | 551.98 | | | | | | | | | | |
| Krypton-88 | | | | | | mBq | | | | 20.83 | | | | | | | | | | |
| Krypton-89 | | | | | | mBq | | | | 8.71 | | | | | | | | | | |
| Lindane | | | | | | µg | | | | 3.44 | | | | | | | | | | |
| Linuron | | | | | | µg | | | | 1.75 | | | | | | | | | | |
| Lithium | | | | | | Pg | | | | 22.38 | | | | | | | | | | |
| m-Cresol | | | | | | Pg | | | | 0.00 | | | | | | | | | | |
| m-Xylene | | | | | | µg | | | | 8.93 | | | | | | | | | | |
| Magnesium | | | | | | µg | | | | 207.11 | | | | | | | | | | |
| Mancozeb | | | | | | µg | | | | 7.66 | | | | | | | | | | |
| Methane, bromo-, Halon 1001 | | | | | | Pg | | | | 996.50 | | | | | | | | | | |
| Permethrin | | | | | | Pg | | | | 115.89 | | | | | | | | | | |
| Phenanthrene | | | | | | Ng | | | | 14.05 | | | | | | | | | | |
| Phenol | | | | | | µg | | | | 5.50 | | | | | | | | | | |

**Table A10**

| **Emissions of various pollutants to Raw from Wheat straw biomass pellet production** | | | |
| --- | --- | --- | --- |
| **Substance** | **Unit** | **Total** |  |
| Antimony | kg Sb eq | 3.59E-17 |  |
| Argon | kg Sb eq | 5.43E-12 |  |
| Barite | kg Sb eq | 4.74E-08 |  |
| Borax | kg Sb eq | 8.56E-11 |  |
| Bromine | kg Sb eq | 3.06E-08 |  |
| Cadmium | kg Sb eq | 4.38E-07 |  |
| Calcite | kg Sb eq | 1.51E-12 |  |
| Cerium | kg Sb eq | 4.61E-18 |  |
| Chromium | kg Sb eq | 8.34E-08 |  |
| Cobalt | kg Sb eq | 2.23E-13 |  |
| Gas, natural/m3 | kg Sb eq | 0.000416 |  |
| Gold | kg Sb eq | 1.28E-07 |  |
| Gold, Au 4.3E-4%, in ore | kg Sb eq | 2.51E-08 |  |
| Gold, Au 4.9E-5%, in ore | kg Sb eq | 1.26E-07 |  |
| Gold, Au 6.7E-4%, in ore | kg Sb eq | 1.34E-07 |  |
| Gold, Au 7.1E-4%, in ore | kg Sb eq | 6.21E-08 |  |
